# Personalizing chronotherapy of immune checkpoint blockade

**DOI:** 10.1136/jitc-2025-013026

**Published:** 2025-10-31

**Authors:** Seline Ismail-Sutton, Bethan Hughes, Zachary S Buchwald, Nicholas I Wreglesworth, Robert Dallmann, Pasquale F Innominato

**Affiliations:** 1 Ysbyty Gwynedd, Bangor, UK; 2 Warwick Medical School and Cancer Research Centre, University of Warwick, Coventry, UK; 3 Acute and Critical Care Medicine, Ysbyty Gwynedd, Bangor, UK; 4 School of Medical and Health Sciences, Bangor University, Bangor, Wales, UK; 5 Radiation Oncology, Emory University Winship Cancer Institute, Atlanta, Georgia, USA; 6 Oncology Department, Ysbyty Gwynedd, Bangor, UK; 7 Division of Biomedical Sciences, Warwick Medical School and SBIDER, University of Warwick, Coventry, England, UK

**Keywords:** Immune Checkpoint Inhibitor, Immunotherapy

## Abstract

Emerging evidence highlights the critical role of time-of-day (ToD) in immunotherapy, with large-scale retrospective studies showing that administering immune checkpoint inhibitors (ICIs) earlier in the day is associated with significantly improved efficacy across all cancer types. This observation aligns with our growing understanding of the circadian system, our internal biological clock, which governs a range of physiological processes, including immune regulation, over a 24-hour scale. Each individual possesses a distinct pace in their circadian rhythm, known as their chronotype, which reflects their natural preference for morning or evening activity, performance and rest. Chronotype has already proven to be a valuable predictor of outcomes in other areas of healthcare, and its application in immuno-oncology holds promising potential. In this commentary, we propose leveraging chronotype as a low-cost and minimally invasive strategy to personalize the timing of ICI administration and enhance therapeutic effectiveness. Despite encouraging retrospective data on ToD effects, current treatment protocols remain largely time-agnostic, hindered by the scantiness of prospective, chronotype-informed clinical trials. We outline here the key steps required to validate and implement chronotype-based scheduling, including rigorous clinical-translational studies. Embracing this temporal dimension could represent a transformative shift toward more precise, personalized, and effective circadian-based cancer immunotherapy.

 There is increasingly compelling evidence, both clinical and mechanistic, that early time-of-day (ToD) administration of immune checkpoint inhibitors (ICI) is associated with improved clinical outcomes, including longer progression-free survival (PFS) and overall survival (OS) across several cancer types.^1^ Yet, despite positive findings in the first prospective randomized clinical trial data,^2^ there are open questions that need addressing with pragmatic prospective chrono-immunotherapy trials.^3^

Chrono-immunotherapy aims to optimize the efficacy of clinically available ICIs by aligning the treatment with the body’s biological clock. This endogenous circadian timing system is regulated by the suprachiasmatic nucleus (SCN) in the hypothalamus, the central pacemaker that coordinates all clocks throughout the body and a wide range of physiological processes, including sleep-wake cycles, hormone secretion, and immune function. Glucocorticoids, for example, not only display a robust clock-dependent rhythm with a strong morning peak but also regulate the diurnal variation of both innate and adaptive immunity.^4^

Exposure to light and social cues, among others, helps entrain and synchronize the body clock to the environment.^1^ Mechanistic studies have demonstrated that circadian rhythms play a critical role in carcinogenesis, metastasis, and antitumor immune responses.^3^ Notably, earlier studies on ToD drug effects primarily involved agents with short half-lives, typically up to 6 hours, whereas ICIs have significantly longer half-lives, lasting several weeks.^3^ While the exposure-response relationship in ICIs is rather complex, baseline clearance is potentially a promising predictor of survival in some ICIs.^5 6^ However, the observed ToD-dependent differences in ICI efficacy could not be attributed to daily changes in clearance despite well-known rhythms in glomerular filtration rate, for example, but instead are thought to be due to the circadian regulation of immune system activity, particularly adaptive immune cells trafficking and activation, and its influence on anticancer responses.^4^ In animal models of colorectal, lung and skin cancer, treatment with anti-PD-L1 (Programmed cell death-ligand 1) therapy at the beginning of the behavioral active phase—compared with the rest phase—elicited a greater antitumor immune response measured by a larger increase in intratumor CD8+T and myeloid-derived suppressor cells. The effect was abolished in animals lacking a circadian clock.^4^ These findings suggest a possible mechanism for the observed improved efficacy of morning ICI administration found in clinical human studies and emphasize the role of the circadian regulation of the immune system in improving ICI efficacy.

With the exception of preliminary reports,^2^ the evidence for ToD variation in ICI efficacy has been derived from retrospective studies.^3^ While these findings are promising, retrospective analyses are inherently susceptible to biases and confounding factors. For instance, healthier patients may be more likely to schedule earlier infusion times, and there is often inconsistency in how “morning” is defined across studies.^3^ These limitations demonstrate the difficulty in drawing definitive conclusions from retrospective data alone.

Therefore, as already highlighted, there is a clear need for well-designed, pragmatic, prospective randomized controlled trials to rigorously evaluate the impact of ToD on PFS and OS, before chrono-immunotherapy can potentially be implemented into clinical practice.^3^ We argue that it is key that the well-established aspect of diurnal preference, or “chronotype” is not overlooked,^7^ expanding the concept of personalized and precision medicine. Measuring and stratifying patients according to their chronotype in ICI chrono-immunotherapy trials has not previously been considered, but the sizeable interindividual differences in circadian regulation have been shown to be important for chronotherapy.^1^

The chronotype of a person is their natural preference for beginning their active phase at a certain ToD. In contrast to the—mostly inbred—mouse strains used in translational studies, however, there are large interindividual differences in human circadian systems and, thus, chronotypes.^7^ The speed of an individual’s circadian clock is largely genetically determined, with significant contributions attributable to behaviors, diseases and medications.^7^ Importantly, the speed of the clock determines the timing of synchronization with the environmental day/night cycle, that is, the phase of entrainment. Faster clocks typically lead to individuals with earlier bed and wake times, while individuals with slower clocks tend to have a later diurnal preference.^8^ Thus, chronotype can significantly shift the timing of behavior and physiology by many hours among different individuals. Questionnaire data, for example, suggest that at least 30% of the population have chronotypes that differ by more than 3 hours from the median.^7^ Therefore, some patients with cancer receiving ICI could potentially feel most alert, active and performant in the mornings, while others do so late in the day or at night, with implications for time of sleep and feeding patterns, all relevant to ICI efficacy.^1^

Establishing chronotype for the purpose of patient stratification is potentially an important variable for the clinical success of chrono-immunotherapy. Below, we discuss how to tackle chronotyping in pragmatic chrono-immunotherapy clinical trials, using patient-generated data and recent advances towards biomolecular markers. The gold standard for the assessment of SCN phase is the dim light melatonin onset (DLMO). Along with the well-characterized cortisol rhythm, a bona fide phase marker of the central biological clock in the SCN and, thus, chronotype.^8^ It is, however, challenging to measure these biomolecular parameters at scale in experimental studies, not to mention clinical practice. Therefore, proxy measurements have been established to include several validated questionnaires such as the Morningness-Eveningness Questionnaire and the Munich Chronotype Questionnaire (MCTQ).^7^ As patient-reported measures, these questionnaires often evaluate the diurnal preference in activity and rest more as a psychological trait than a biological construct.

A less subjective approach is to use wearable biosensors, which do not require active patient engagement (besides wearing the device). These can continuously and remotely monitor parameters useful as phase markers of the circadian clock, such as locomotor activity, body temperature or heart rate.^9^ Wearable devices allowing dynamic, multidimensional and longitudinal monitoring are becoming increasingly valuable in oncology for multiple purposes, including improving patient safety, evaluating behaviors and predicting toxicity.^1 9^ In chrono-immunotherapy, they can also provide additional insight towards personalization of treatment based on the individual phase of physiological functions relevant to immunotherapy.

Novel minimally or non-invasive biomolecular tests to estimate circadian clock phase and function from only one or few samples are on the horizon.^8 10^ These approaches harness a prior knowledge of circadian regulation of the transcriptome, proteome or metabolome, and often use machine learning to estimate the circadian phase and/or clock disruption of an individual from one or two biosamples. This might include peripheral blood mononuclear cells, serum, saliva, exhaled breath or hair follicle.^8^ Importantly, while a number of these have been validated against DLMO in healthy volunteers, so far, these have not been tested in clinical cohorts such as patients with cancer.^8^ Previously, however, such algorithmic approaches have been successfully applied to populations of patients, suggesting a high chance of success in estimating individual molecular circadian clock phase from a single snapshot for approaches like TimeTeller.^8 10^

Harnessing chronotype information to improve outcomes has already been successfully demonstrated in the Treatment in Morning versus Evening clinical trial, which assessed if timing antihypertensive medication based on chronotype affected cardiovascular outcomes.^11^ This prospective randomized, blinded trial assessed chronotype using the MCTQ mid-sleep time on free days for corrected sleep debt. The results revealed that evening administration of antihypertensive medications in evening-type patients (“owls” using an ornithological metaphor) with hypertension was associated with a reduced risk of hospitalization and a lower incidence of non-fatal myocardial infarction. In contrast, for morning-type patients (“larks”), this reduced risk was observed when medications were taken in the morning rather than the evening, while individuals with intermediate chronotype did not exhibit differences.^11^ This study emphasizes the potential of using individual chronotype and tailored treatment to maximize patient benefit through optimization of existing resources, and highlights that a one-size-fits-all approach might have resulted in falsely negative pragmatic trials with ultimate detriment to many patients.

In conclusion, we believe that pragmatic chrono-immunotherapy trials ought to include some form of individual assessment of circadian phase. This could minimize the potential confounding difference between biological (internal) time versus “wall clock” ToD. This aspect can be particularly relevant in geographical areas with artificial time zones, where there could be a desynchrony between the social and the sun zeitgeber, as observed, for instance, in the USA, Europe or China.^12^ This approach could additionally lead to dedicated studies for personalized ToD of ICI administration maximizing the potential benefit to each and every patient. This could have significant implications for the future of precision oncology. If treatments are not tailored to an individual’s unique physiology, there is a higher likelihood of decreased efficacy and greater toxicity. To summarize, a patient’s chronotype can be evaluated in several ways, including through validated questionnaires, wearable devices or biomolecular snapshot methods.

Previously, it has been questioned whether chronotherapy is too complex for effective clinical implementation. It is accepted now that most targeted anticancer therapies will only work in a certain portion of patients. Thus, clinical trials have developed towards allowing biomarker-based subgroup identification and using adaptive enrichment or biomarker stratification designs.^13^ Accordingly, if randomized chronotherapy-based trials were conducted, consideration of patient chronotypes should be considered as it could potentially improve the trials’ ability to identify significant advantages in outcomes compared with the whole population. Thus, alongside pragmatism, personalization could be harnessed to help ensure that the right patient receives the right treatment at the right time, for the maximal benefit to all, and that the low-hanging fruit is not rotten.

## Supplementary material

10.1136/jitc-2025-013026 online supplemental file 1
