# Supplementary figures and images for: Personalizing chronotherapy of immune checkpoint blockade
Source: J Immunother Cancer. 2025 Oct 31;13(10):e013026. doi: 10.1136/jitc-2025-013026 (PMC12581061; doi:10.1136/jitc-2025-013026)

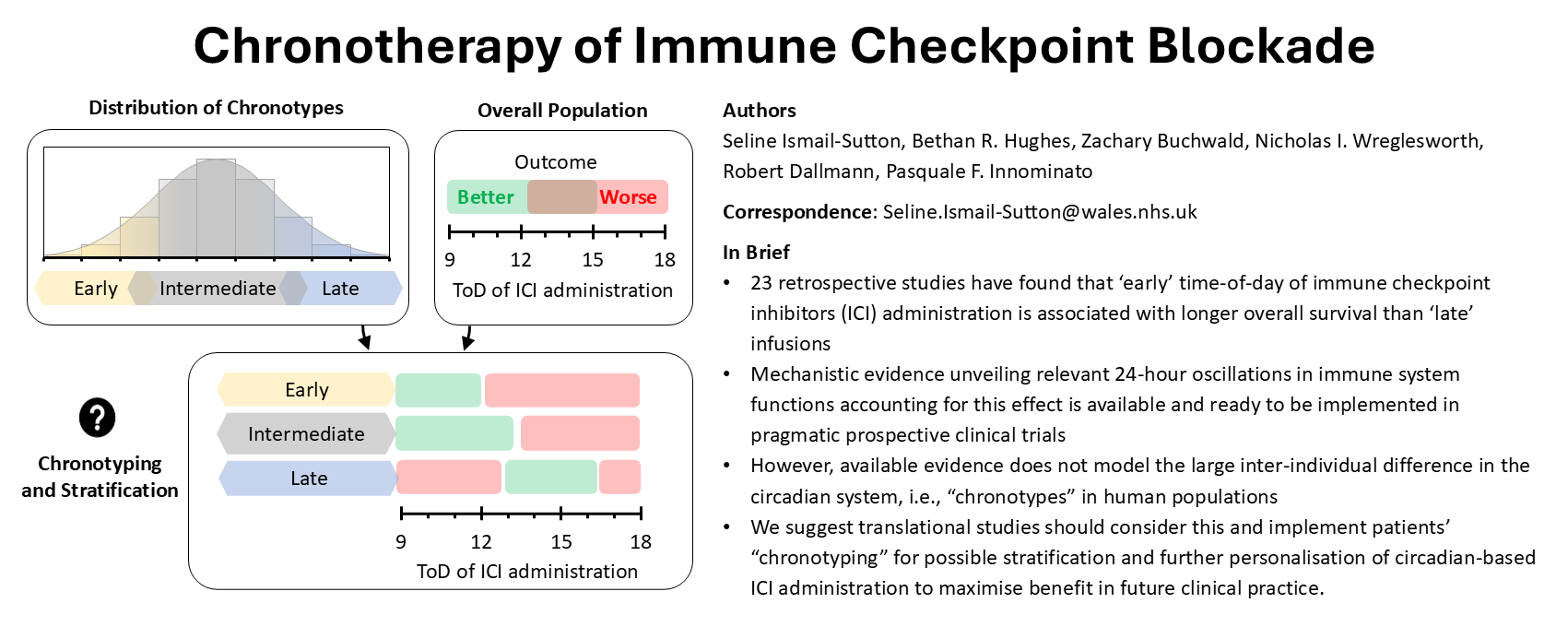

Supplement: online supplemental file 1 [file jitc-13-10-s001.tif]
